# Association of non-insulin-based insulin resistance indices with disease severity and adverse outcome in idiopathic pulmonary arterial hypertension: a multi-center cohort study

**DOI:** 10.1186/s12933-024-02236-9

**Published:** 2024-05-03

**Authors:** Sicheng Zhang, Luyang Gao, Sicong Li, Manqing Luo, Lichuan Chen, Qunying Xi, Zhihui Zhao, Qing Zhao, Tao Yang, Qixian Zeng, Xin Li, Zhihua Huang, Anqi Duan, Yijia Wang, Qin Luo, Yansong Guo, Zhihong Liu

**Affiliations:** 1 Center for Respiratory and Pulmonary Vascular Diseases, National Center for Cardiovascular Diseases, Fuwai Hospital, Chinese Academy of Medical Sciences and Peking Union Medical College, No. 167, Beilishi Road, Beijing, 100037 Xicheng China; 2 Department of Cardiology, Fujian Provincial Hospital, Shengli Clinical Medical College of Fujian Medical University, No. 134, East Street, Gulou District, Fuzhou, 350001 Fujian China; 3 Center for Pulmonary Vascular Diseases, Fuwai Hospital, Chinese Academy of Medical Sciences, Shenzhen, No. 12, Langshan Road, Shenzhen, 518057 Nanshan China

**Keywords:** Idiopathic pulmonary arterial hypertension, Insulin resistance, Metabolic score for insulin resistance, Prognosis, Severity

## Abstract

**Background:**

Insulin resistance (IR) plays an important role in the pathophysiology of cardiovascular disease. Recent studies have shown that diabetes mellitus and impaired lipid metabolism are associated with the severity and prognosis of idiopathic pulmonary arterial hypertension (IPAH). However, the relationship between IR and pulmonary hypertension is poorly understood. This study explored the association between four IR indices and IPAH using data from a multicenter cohort.

**Methods:**

A total of 602 consecutive participants with IPAH were included in this study between January 2015 and December 2022. The metabolic score for IR (METS-IR), triglyceride to high-density lipoprotein cholesterol (TG/HDL-C) ratio, triglyceride and glucose (TyG) index, and triglyceride-glucose-body mass index (TyG-BMI) were used to quantify IR levels in patients with IPAH. The correlation between non-insulin-based IR indices and long-term adverse outcomes was determined using multivariate Cox regression models and restricted cubic splines.

**Results:**

During a mean of 3.6 years’ follow-up, 214 participants experienced all-cause death or worsening condition. Compared with in low to intermediate-low risk patients, the TG/HDL-C ratio (2.9 ± 1.7 vs. 3.3 ± 2.1, P = 0.003) and METS-IR (34.5 ± 6.7 vs. 36.4 ± 7.5, P < 0.001) were significantly increased in high to intermediate-high risk patients. IR indices correlated with well-validated variables that reflected the severity of IPAH, such as the cardiac index and stroke volume index. Multivariate Cox regression analyses indicated that the TyG-BMI index (hazard ratio [HR] 1.179, 95% confidence interval [CI] 1.020, 1.363 per 1.0-standard deviation [SD] increment, P = 0.026) and METS-IR (HR 1.169, 95% CI 1.016, 1.345 per 1.0-SD increment, P = 0.030) independently predicted adverse outcomes. Addition of the TG/HDL-C ratio and METS-IR significantly improved the reclassification and discrimination ability beyond the European Society of Cardiology (ESC) risk score.

**Conclusions:**

IR is associated with the severity and long-term prognosis of IPAH. TyG-BMI and METS-IR can independently predict clinical worsening events, while METS-IR also provide incremental predictive performance beyond the ESC risk stratification.

**Supplementary Information:**

The online version contains supplementary material available at 10.1186/s12933-024-02236-9.

## Introduction

Pulmonary arterial hypertension (PAH) is characterized by the progressive elevation of pulmonary artery pressure and pulmonary vascular resistance, ultimately resulting in right heart failure and even mortality. It represents an advanced stage of diverse cardiovascular and pulmonary ailments. Among patients with PAH, those with idiopathic pulmonary arterial hypertension (IPAH) have the highest proportion and the worst prognosis, imposing a heavy economic burden of the disease [1]. Recently, diabetes mellitus (DM) has emerged as a novel phenotype-associated cardiopulmonary complication of PAH [2]. The prevalence of impaired glucose tolerance, DM, and abnormal blood lipid metabolism is considerably higher in patients with IPAH than in the general population [3, 4]. Moreover, patients with IPAH and concurrent glucose abnormalities or lipid metabolism disorders have a worse prognosis than their counterparts without these complications [5, 6]. This implies that metabolic diseases may diminish the response to targeted medications in patients with IPAH [7].

Insulin resistance (IR) refers to the diminished sensitivity or impaired response of target organs or tissues to insulin, which culminates in the compromised uptake and utilization of glucose [8]. IR has been established as a canonical risk factor for several cardiovascular diseases (CVDs), such as coronary artery disease, stroke, and peripheral vascular disease [9]. The hyperinsulinemic–euglycemic clamp technique is considered to be the benchmark for quantifying IR; however, its technical complexity and high cost pose challenges for its widespread implementation [10]. Therefore, alternative indices, including the triglyceride and glucose (TyG) index, triglyceride–glucose–body mass index (TyG-BMI), triglyceride-to-high-density lipoprotein cholesterol (TG/HDL-C) ratio, and metabolic score for IR (METS-IR), have been shown to be significant, effective, and feasible measures for evaluating IR [11–14].

Previous studies have established a relationship between IR indices and an elevated probability of all-cause mortality and readmission in patients with heart failure [15, 16]. Moreover, various studies have demonstrated a correlation of IR with various cardiovascular ailments, such as stroke, atherosclerosis, coronary artery lesions, and cardiovascular incidents in post-percutaneous coronary intervention patients [17–20].

However, research on the correlation between different IR indicators and the severity of IPAH is scarce, and no study has assessed the predictive efficacy of these IR indicators in relation to clinical deterioration events in IPAH. Therefore, we conducted a multi-center retrospective cohort study to investigate the association between the four IR indicators and functional status, ultrasound indicators, hemodynamic parameters, and adverse outcomes in patients with IPAH. In addition, we evaluated whether the inclusion of IR indices in existing risk stratification tools could supplement predictive efficacy. These findings would have significant implications for clinicians in terms of promptly identifying and treating patients with high-risk IPAH.

## Methods

### Study design and population

This multi-center retrospective cohort study was performed at three level-3 hospitals in China: Fuwai Hospital of Chinese Academy of Medical Sciences (Beijing), Fujian Provincial Hospital (Fuzhou), and Fuwai Hospital (Shenzhen). In total, 694 consecutive patients diagnosed or treated with IPAH were recruited between January 2015 and December 2022. The baseline assessment included data acquired at the time of IPAH diagnosis in incident cases (diagnosed after January 1, 2015) and data obtained at the most recent visit in previously treated patients (diagnosed before January 1, 2015).

The diagnosis of IPAH was established by confirming precapillary pulmonary hypertension (elevated mean pulmonary artery pressure [mPAP] > 20 mmHg and pulmonary artery wedge pressure [PAWP] ≤ 15 mmHg) with elevated pulmonary vascular resistance (PVR > 2 wood units) [2]. Patients with findings typical of left heart disease, significant lung disease, chronic thromboembolic pulmonary hypertension, or other known causes of PAH were classified as having other types of pulmonary hypertension according to the European Society of Cardiology (ESC) guidelines, and thus were not included in the study. The date of diagnosis was defined as the date of the first right heart catheterization that met the hemodynamic criteria for pre‑capillary pulmonary hypertension. After excluding confusing or missing data, 602 participants were finally included in the final statistical analysis that investigated the relationship between IR and the severity and prognosis of IPAH, (Additional file 1: Fig. S1). The study protocol conformed to the ethical guidelines of the Declaration of Helsinki and was revised and approved by the local ethics committee. Written informed consent was obtained from all patients.

### Measurements and definitions

Demographic data, medical history, and clinical parameters were recorded upon admission. Fasting peripheral venous blood samples were collected before right heart catheterization (RHC) to obtain laboratory data, including fasting plasma glucose (FPG), triglyceride (TG), total cholesterol, low-density lipoprotein cholesterol (LDL-C), high-density lipoprotein cholesterol (HDL-C), levels of serum N-terminal pro-brain natriuretic peptide (NT-proBNP), and serum creatinine levels. Echocardiography was completed within 48 h of admission. RHC, cardiopulmonary exercise testing (CPET), and pulmonary function tests (PFT) were performed when the patients were in a stable condition. All three centers set the transducer to zero level at the mid-thoracic line (the intersection of the frontal plane at the mid-thoracic level, transverse plane at the level of fourth anterior intercostal space, and midsagittal plane) and at the level of the left atrium (the patient in a supine position, halfway between the anterior sternum and bed surface) [21, 22]. After the sheath was inserted, the patient was allowed to rested briefly to reach a stable state before measuring hemodynamic parameters, ensuring standardization of RHC procedures [23, 24]. Additionally, CPET was conducted using an upright cycle ergometer (COSMED, Rome, Italy). Patients rested on the machine for 3 min and then pedaled without workload for another 3 min. The work rate was gradually increased (5 to 30 W/min) based on the individual’s estimated exercise tolerance until exhaustion or symptom limitation. Standardized spreadsheets were designed to collect retrospective data. All three centers followed the treatment strategies recommended by the ESC guidelines and developed targeted drug therapy plans based on the patient's condition [2, 25, 26].

DM was defined as a previous diagnosis of any type of diabetes, being on treatment approved for DM, fasting blood glucose levels ≥ 126 mg/dL (7.0 mmol/L) documented on 2 different days, or blood glucose levels ≥ 200 mg/dL (11.1 mmol/L) at the 120 minute time-point of the oral glucose tolerance test, hemoglobin A1c (HbA1c) level ≥ 6.5%, or in a patient with classic symptoms of hyperglycemia or hyperglycemic crisis, a random plasma glucose level ≥ 200 mg/dL (11.1 mmol/L) [27]. Hypertension was diagnosed based on self-reported physician diagnosis, recent use of an antihypertensive agent, or blood pressure ≥ 140/90 mmHg [28]. The formulae used to calculate the four IR indices [14, 29–31] are summarized in Additional file 1: Table S1.

The severity of IPAH was assessed at baseline using parameters recommended by the ESC[2]: World Health Organization Functional Class (WHO-FC); 6 min walking distance (6MWD); NT‑proBNP levels; peak oxygen uptake (peak VO_2_); echocardiography parameters, including pericardial effusion and tricuspid annular plane systolic excursion (TAPSE)/systolic pulmonary arterial pressure (sPAP); hemodynamic parameters, including right atrial pressure (RAP), venous oxygen saturation (S_V_O_2_), stroke volume index (SVI) and cardiac index.

Patients were classified as low, intermediate-low, intermediate-high, or high risk based on the risk stratification strategy recommend by the ESC [2]. One to four points were assigned to each parameter in this prediction model, which included WHO-FC, 6MWD, and NT-proBNP (details are shown in Additional file 1: Table S2). The risk score for each individual was determined by dividing the sum of all grades by the number of variables and rounding to the next integer.

### Endpoints and follow-up

Clinical worsening, the primary outcome of this study, was defined as the first occurrence of any of the following events: all-cause death, lung transplantation, or re-hospitalization because of heart failure (including right, left, or whole heart failure). Follow-up data were obtained through outpatient clinical visits, readmission, or telephonic interviews with the patients or their relatives after discharge. The endpoints were adjudicated by an independent committee.

### Statistical analysis

Continuous variables were presented as the mean ± standard deviation (SD) or median [25th–75th percentile] and analyzed using Student’s t test (normally distributed) or the Mann–Whitney U test (nonnormally distributed). The chi-squared test or Fisher’s exact test was used to compare categorical variables as counts (percentages). Correlations between the IR indices and established markers of PAH severity were examined using Pearson, Spearman, or Point–Biserial correlation coefficients. One-way analysis of variance was used to compare differences among different risk strata. Restricted cubic spline curves were used to evaluate the relationship between IR indices and clinical worsening.

The four IR indices were standardized (Z-score) and added to the unadjusted or adjusted models to evaluate the influence of a 1.0-standard deviation (SD) increment in the indices on clinical worsening. Univariate Cox regression analysis was performed to identify risk factors for clinical worsening, and factors with P < 0.05 or clinical significance were retained in the multivariable Cox regression model. To exclude confounding factors, Model 1 was adjusted for age, sex, and ethnicity. Model 2 was adjusted for factors in Model 1 plus DM, WHO-FC, 6MWD, ln (NT-proBNP), HbA1c, and PAH-specific medications. Model 3 was adjusted for factors in Model 2 plus S_V_O_2_, cardiac index, and PVR. The variance inflation factor (VIF) method was used to test for collinearity; no clear evidence of multicollinearity was found in the overall population (VIF for the included variables were < 5). Subgroup analyses, stratified by sex, age, body mass index (BMI), and DM were performed to determine the interaction effects, and the P value for the interaction was calculated.

Receiver operating characteristic (ROC) curves were used to predict clinical worsening based on the ESC risk score, and the area under the curve, as measured by the C-statistic, was computed to quantify the predictive power for clinical worsening. Additionally, the net reclassification improvement (NRI) and integrated discrimination improvement (IDI) indices were calculated to assess the predictive value of the four indices further based on the established risk stratification score.

Statistical significance was set at P < 0.05 (two‐sided). Data analyses were performed using R-studio (version 4.2.2; R Foundation for Statistical Computing, Vienna, Austria).

## Results

### Baseline characteristics

Overall, 602 consecutive patients with IPAH (median age, 32 [26.0–39.0] years; 77.2% female; 97.0% Han Chinese) were included in this study. Among 191 patients diagnosed previously, 47 (24.6%) received PAH combination therapy, with a median time from diagnosis to enrollment of 1.5 [0.5–3.0] years. During the follow-up period, 214 (35.5%) patients experienced clinical worsening. The baseline characteristics of the participants of those who did and those who did not experience clinical worsening are presented in Table 1. In brief, compared with patients without worsening, those with clinical worsening tended to be of non-Han ethnicity and had worse WHO-FC, more restricted 6MWD, higher rates of diabetes, and pericardial effusion. Approximately 91.0% of the participants received targeted therapy for PAH during the initial hospitalization. The remaining patients refused to accept this treatment, mainly because of the financial burden, intolerability, and caution regarding adverse effects. Compared with patients without clinical worsening, BMI, NT-proBNP, TG, RAP, and PVR were significantly higher, whereas HDL-C, FPG, peak VO_2_, TAPSE/sPAP, cardiac index, SVI, and S_V_O_2_ were lower in patients with than in those without clinical worsening. Patients with clinical worsening also had a substantially higher TyG-BMI index, TG/HDL-C ratio, and METS-IR than those without clinical worsening. No significant difference in the TyG index was found between the two groups. Owing to the retrospective study design, 14.3% (n = 86) of patients had CPET and PFT data missing, and the rest of the variables in Table 1 were complete.

**Table 1:** Baseline characteristics of study population

Variables	Non-CW (*n* = 388)	CW (*n* = 214)	*P* value
Demographics			
Age, years	33.0 [26.0–40.0]	32.0 [27.0–38.0]	0.539
Female, *n* (%)	305 (78.6)	160 (74.8)	0.330
Han ethnicity, *n* (%)	381 (98.2)	203 (94.9)	0.040
BMI, kg/m^2^	22.6 [20.6–25.0]	23.0 [20.4–26.4]	0.042
Current smoking, *n* (%)	39 (10.1)	27 (12.6)	0.408
Previously diagnosed, *n* (%)	127 (32.7)	64 (29.9)	0.476
Clinical evaluation and comorbidities			
WHO-FC, *n* (%)			< 0.001
I or II	230 (59.3)	91 (42.5)	
III or IV	158 (40.7)	123 (57.5)	
6MWD, m	432 [375–495]	394 [315–464]	< 0.001
Diabetes mellitus, *n* (%)	44 (11.3)	39 (18.2)	0.026
Arterial hypertension, *n* (%)	47 (12.1)	30 (14.0)	0.587
Coronary heart disease, *n* (%)	4 (1.0)	6 (2.8)	0.103
Atrial fibrillation, *n* (%)	8 (2.1)	5 (2.3)	0.824
COPD, *n* (%)	8 (2.1)	6 (2.8)	0.563
Borderline PAWP (12–15 mmHg), *n* (%)	44 (11.3)	35 (16.4)	0.081
Low DL_CO_(< 45% pred), *n* (%)	33 (9.9)	15 (8.2)	0.503
Laboratory data			
NT-proBNP, pg/mL	784.4[241.2–1745.0]	1454.8[928.0–2393.0]	< 0.001
FPG, mmol/L	5.0 [4.6–5.4]	4.9 [4.5–5.3]	0.015
HbA1c, %	5.7 [5.3, 6.1]	5.9 [5.6, 6.4]	< 0.001
Albumin, g/L	44.6 [41.2–47.8]	42.5 [39.3–46.0]	< 0.001
ALT, IU/L	23.0 [15.0–34.0]	25.0 [18.0–39.0]	0.042
AST, IU/L	26.0 [21.0–34.0]	26.0 [21.0–35.0]	0.781
Triglyceride, mmol/L	1.2 [0.9–1.7]	1.3 [0.9–1.8]	0.040
Cholesterol, mmol/L	4.2 ± 0.8	4.1 ± 1.1	0.158
HDL-C, mmol/L	1.2 [1.0–1.3]	1.1 [0.9–1.2]	< 0.001
LDL-C, mmol/L	2.6 [2.2–3.0]	2.6 [2.0–3.2]	0.617
Serum creatinine, umol/L	71.0 [63.0–81.0]	73.8 [62.1–84.4]	0.061
CPET and PFT			
peak VO_2_, mL/min/kg	13.3 [10.8–16.1]	11.6 [9.6–13.7]	< 0.001
FEV1, % pred	80.0 [71.3–87.1]	81.0 [72.0–90.0]	0.409
DL_CO_, % pred	62.0 [52.0–71.6]	63.9 [53.5–74.0]	0.309
Echocardiography			
Pericardial effusion, *n* (%)	55 (14.2)	51 (23.8)	0.004
TAPSE, mm	16.0 [13.0–19.0]	15.0 [13.0–17.0]	0.006
sPAP, mmHg	87.0 [74.0–102.0]	91.0 [77.0–107.0]	0.038
TAPSE/sPAP, mm/mmHg	0.2 [0.1–0.2]	0.2 [0.1–0.2]	0.011
LA, mm	29.0 [27.0–32.0]	30.0 [27.0–32.0]	0.796
LVED, mm	37.0 [33.0–41.0]	35.0 [32.0–38.8]	< 0.001
RVED, mm	31.5 [27.2–36.0]	34.0 [30.0–40.0]	< 0.001
Right heart catheterization			
RAP, mmHg	4.0 [2.0–7.0]	5.0 [2.0–8.0]	0.011
Cardiac index, L/min/m^2^	2.9 [2.3–3.5]	2.7 [2.2–3.2]	0.020
SVI, mL/m^2^	34.4 [27.8–44.5]	31.6 [24.8–40.2]	0.002
S_V_O_2_, %	71.0 [65.7–75.3]	69.7 [65.0–73.7]	0.020
mPAP, mmHg	54.0 [46.0–63.2]	55.0 [47.0–68.0]	0.120
PAWP, mmHg	7.0 [5.0–9.0]	7.0 [4.0–10.0]	0.905
PVR, wood units	11.7 [8.1–15.8]	12.9 [9.5–16.3]	0.010
Treatment			
PAH‑specific treatment, *n* (%)	350 (90.2)	198 (92.5)	0.422
PAH combination therapy, *n* (%)	157 (41.0)	57 (27.3)	< 0.001
Parenteral prostacyclin, *n* (%)	32 (8.2)	26 (12.2)	0.120
Statins, *n* (%)	15 (3.9)	12 (5.6)	0.323
Fenofibrate, *n* (%)	6 (1.5)	3 (1.4)	0.889
Metformin, *n* (%)	34 (8.8)	22 (10.3)	0.540
Insulin, *n* (%)	15 (3.9)	12 (5.6)	0.323
Acarbose, *n* (%)	6 (1.5)	9 (4.2)	0.045
DPP‑4 inhibitors, *n* (%)	5 (1.3)	2 (0.9)	0.698
SGLT‑2 inhibitors, *n* (%)	5 (1.3)	3 (1.4)	0.908
Insulin resistance indices			
TyG index	8.5 ± 0.5	8.5 ± 0.5	0.224
TyG-BMI index	194.0 ± 33.7	202.0 ± 39.8	0.011
TG/HDL-C ratio	2.9 ± 1.8	3.5 ± 2.2	< 0.001
METS-IR	34.5 ± 6.6	37.2 ± 7.9	< 0.001

Data are presented as mean ± standard deviation, median [25th–75th percentile] or number (percentage)

*ALT* alanine aminotransferase, *AST* aspartate aminotransferase, *BMI* body mass index, *COPD* chronic obstructive pulmonary disease, *CPET* cardiopulmonary exercise testing, *CW* clinical worsening, *DL*_*CO*_ carbon monoxide diffusing capacity, *FEV1* forced expiratory volume in one second, *FPG* fasting plasma glucose, *HbA1c* hemoglobin A1c, *HDL-C* high-density lipoprotein cholesterol, *LA* left atrium dimension, *LDL-C* low-density lipoprotein cholesterol, *LVED* left ventricular end-diastolic diameter, *METS-IR* metabolic score for insulin resistance, *mPAP* mean pulmonary arterial pressure, *NT-proBNP* N-terminal pro-brain natriuretic peptide, *PAH* pulmonary arterial hypertension, *PAWP* pulmonary arterial wedge pressure, *PFT* pulmonary function test, *PVR* pulmonary vascular resistance, *RAP* right atrial pressure, *RVED* right ventricular end-diastolic diameter, *6MWD* 6-min walk distance, *sPAP* systolic pulmonary arterial pressure, *S*_*V*_*O*_*2*_ mixed venous oxygen saturation, *SVI* stroke volume index, *TAPSE* tricuspid annular plane systolic excursion, *TG/HDH-C* triglyceride to high-density lipoprotein cholesterol ratio, *TyG* Triglyceride and glucose, *TyG-BMI* triglyceride-glucose-body mass index, *VO2* oxygen uptake, *WHO-FC* World Health Organization functional class

### Association between IR indices and severity of IPAH

**Fig. 1:**
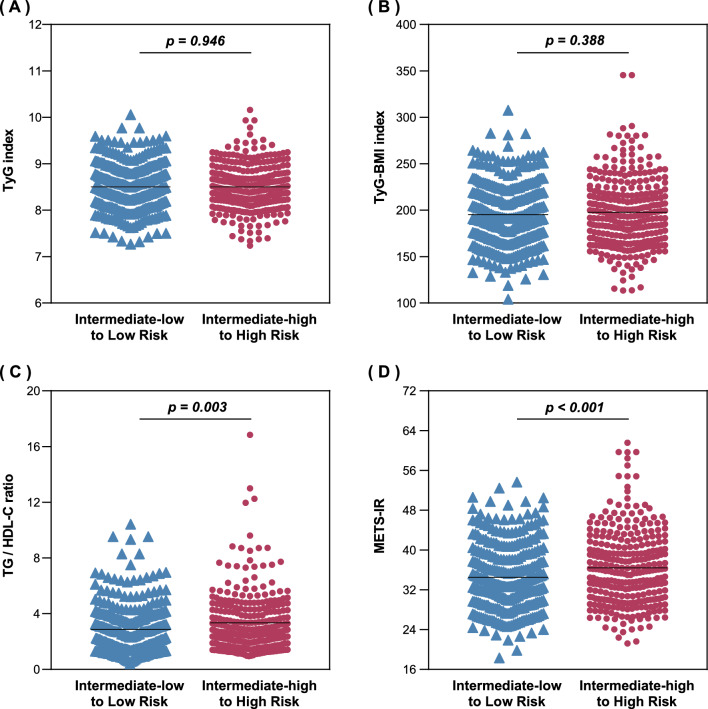
Association between insulin resistance indices and the ESC risk score. Scatterplots of the relationship between risk stratification and TyG index (**A**), TyG-BMI index (**B**), TG/HDL-C ratio (**C**) and METS-IR (**D**). *ESC* European Society of Cardiology, *METS-IR* metabolic score for insulin resistance, *TG/HDH-C* triglyceride to high-density lipoprotein cholesterol ratio, *TyG* Triglyceride and glucose, *TyG-BMI* triglyceride glucose-body mass index

As shown in Table 2, different indices were associated with different indicators of IPAH severity. For instance, the METS-IR was mildly correlated with peak VO_2_, RAP, cardiac index, SVI, and S_V_O_2_. However, no correlations were observed between METS-IR and WHO-FC (r = 0.018, P = 0.734), 6MWD (r = -0.034, P = 0.518), NT-proBNP (r = 0.073, P = 0.160), pericardial effusion (r = 0.093, P = 0.075), or TAPSE/sPAP (r = 0.085, P = 0.104). In addition, Fig. 1 shows the levels and distribution of the four indices in the different groups, using the risk stratification recommended by the ESC guidelines. The TG/HDL-C ratio (low to intermediate-low risk vs. high to intermediate-high risk: 2.9 ± 1.7 vs. 3.3 ± 2.1, P = 0.003) and METS-IR (low to intermediate-low risk vs. high to intermediate-high risk: 34.5 ± 6.7 vs. 36.4 ± 7.5, P < 0.001) were increased as the ESC risk score escalated.

**Table 2:** Correlation analysis between insulin resistance indices with established markers of PAH severity

Variables	TyG index		TyG-BMI index		TG/HDL-C ratio		METS-IR	
	Coefficient (*r*)	*P* value	Coefficient (*r*)	*P* value	Coefficient (*r*)	*P* value	Coefficient (*r*)	*P* value
WHO-FC	−0.034	0.518	−0.027	0.600	0.056	0.285	0.018	0.734
6MWD	0.079	0.128	0.034	0.521	−0.035	0.508	−0.034	0.518
ln (NT-proBNP)	−0.011	0.840	−0.045	0.393	0.132	0.011	0.073	0.160
peak VO_2_	−0.019	0.717	−0.191	< 0.001	−0.165	0.001	−0.276	< 0.001
Pericardial effusion	−0.042	0.420	0.016	0.765	−0.041	0.428	0.093	0.075
TAPSE/sPAP	0.075	0.150	0.177	0.001	0.030	0.564	0.085	0.104
RAP	−0.063	0.229	0.075	0.150	0.029	0.577	0.171	0.001
cardiac index	−0.141	0.007	−0.134	0.010	−0.228	< 0.001	−0.229	< 0.001
SVI	−0.135	0.009	−0.145	0.005	−0.228	< 0.001	−0.239	< 0.001
S_V_O_2_	−0.045	0.391	−0.102	0.051	−0.192	< 0.001	−0.233	< 0.001

*6MWD* 6-min walking distance, *METS-IR* metabolic score for insulin resistance, *NT-proBNP* N-terminal pro-brain natriuretic peptide, *PAH* pulmonary arterial hypertension, *RAP* right atrial pressure, *sPAP* systolic pulmonary arterial pressure, *S*_*V*_*O*_*2*_ mixed venous oxygen saturation, *SVI* stroke volume index, *TAPSE* tricuspid annular plane systolic excursion, *TG/HDH-C* triglyceride to high-density lipoprotein cholesterol ratio, *TyG* Triglyceride and glucose, *TyG-BMI* triglyceride-glucose-body mass index, *VO*_*2*_ oxygen uptake, *WHO-FC* World Health Organization functional class

### Relationship between IR indices and adverse outcome of IPAH

**Fig. 2:**
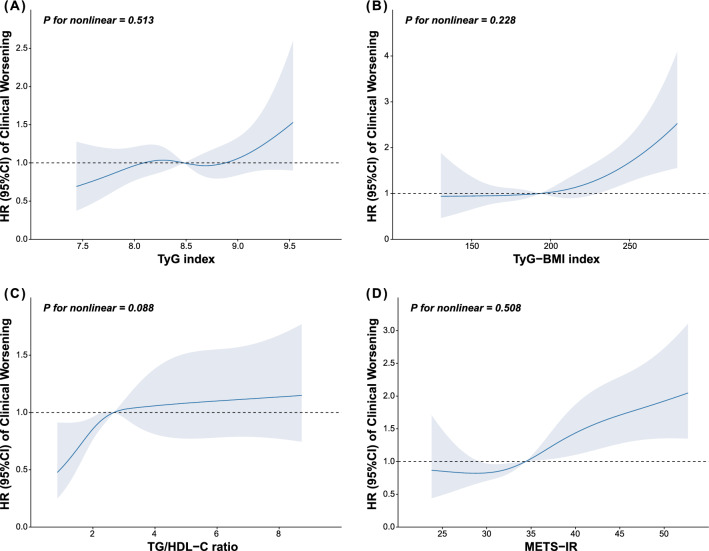
Hazard ratios of clinical worsening as a function of baseline insulin resistance indices. Insulin resistance indices as continuous variables fitted an unadjusted COX regression model using restricted cubic spline regression. *CI* confidence interval, *HR* hazard ratio, *METS-IR* metabolic score for insulin resistance, *TG/HDH-C* triglyceride to high-density lipoprotein cholesterol ratio, *TyG* Triglyceride and glucose, *TyG-BMI* triglyceride glucose-body mass index

During a mean follow-up period of 3.6 years, 214 (35.5%) patients experienced primary endpoint event. We defined the four indices as continuous variables with the median as the reference point and used restricted cubic spline regression to fit the unadjusted Cox proportional hazards model. Unadjusted spline plots showed no nonlinear association between the four indices and the hazard ratio (HR) for clinical worsening (Fig. 2). To evaluate the predictive value of IR indices for clinical worsening further, we established three Cox regression models to assess the impact of a 1.0-SD increment in the four IR indices on endpoint events (Table 3). In the unadjusted Cox regression models, TyG-BMI, TG/HDL-C ratio, and METS-IR were associated with adverse outcomes. After fully adjusting for covariates in Model 3, we observed that only the TyG-BMI index (HR 1.179, 95% confidence interval [CI] 1.020, 1.363 per 1.0-SD increment, P = 0.026) and METS-IR (HR 1.169, 95% CI 1.016, 1.345 per 1.0-SD increment, P = 0.030) independently predicted all-cause death, lung transplantation, and rehospitalization because of heart failure in patients with IPAH. No collinearity problems were detected in the multivariate Cox regression analysis.

**Table 3:** Association between insulin resistance indices and clinical worsening

	Unadjusted		Model 1		Model 2		Model 3	
	HR (95% CI)	*P* value	HR (95% CI)	*P* value	HR (95% CI)	*P* value	HR (95% CI)	*P* value
TyG index	1.130 (0.988, 1.293)	0.074	1.127 (0.983, 1.291)	0.087	1.121 (0.976, 1.288)	0.106	1.117 (0.970, 1.285)	0.123
TyG-BMI index	1.279 (1.118, 1.463)	< 0.001	1.237 (1.083, 1.414)	0.002	1.178 (1.024, 1.355)	0.022	1.179 (1.020, 1.363)	0.026
TG/HDL-C ratio	1.133 (1.019, 1.259)	0.020	1.133 (1.017, 1.263)	0.023	1.050 (0.938, 1.174)	0.400	1.046 (0.933, 1.173)	0.439
METS-IR	1.317 (1.168, 1.485)	< 0.001	1.278 (1.132, 1.443)	< 0.001	1.167 (1.022, 1.331)	0.023	1.169 (1.016, 1.345)	0.030

Model 1: Adjusted for age, gender and ethnicity

Model 2: Adjusted for variables from Model 1 plus diabetes mellitus, WHO-FC, 6MWD, ln (NT-proBNP), HbA1c and PAH‑specific treatment

Model 3: Adjusted for variables from Model 2 plus S_V_O_2_, cardiac index and PVR

*CI* confidence interval, *HbA1c* hemoglobin A1c, *HR* hazard ratio, *ln* logarithmically transformed, *NT-proBNP* N-terminal pro-brain natriuretic peptide, *METS-IR* metabolic score for insulin resistance, *6MWD* 6-min walk distance, *PAH* pulmonary arterial hypertension, *PVR* pulmonary vascular resistance, *S*_*V*_*O*_*2*_ mixed venous oxygen saturation, *TG/HDL-C* triglyceride to high-density lipoprotein cholesterol ratio, *TyG* triglyceride and glucose, *TyG-BMI* triglyceride-glucose-body mass index, *WHO-FC* World Health Organization functional class

**Fig. 3:**
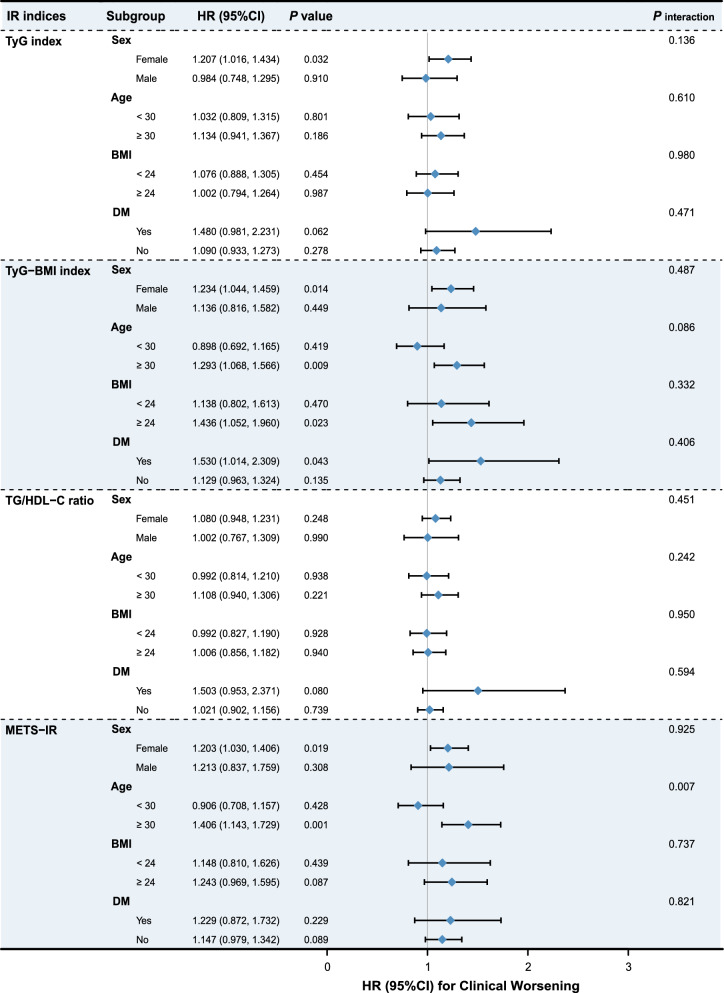
Forest plot of hazard ratios by patient subgroups. Each subgroup was adjusted for age, gender, ethnicity, DM, WHO-FC, 6MWD, ln (NT-proBNP), HbA1c, and PAH‑specific treatment, S_V_O_2_, Cardiac index and PVR. Hazard ratios are presented as per 1.0-SD increase in the insulin resistance indices for clinical worsening. *BMI* body mass index, *CI* confidence interval, *DM* diabetes mellitus, *HbA1c* hemoglobin A1c, *HR* hazard ratio; *ln* logarithmically transformed, *NT-proBNP* N-terminal pro-brain natriuretic peptide, *METS-IR* metabolic score for insulin resistance, *6MWD* 6-min walk distance, *PAH* pulmonary arterial hypertension, *PVR* pulmonary vascular resistance, *S*_*V*_*O*_*2*_ mixed venous oxygen saturation, *TG/HDL-C* triglyceride to high-density lipoprotein cholesterol ratio, *TyG* triglyceride and glucose, *TyG-BMI* triglyceride glucose-body mass index, *WHO-FC* World Health Organization functional class

In addition, we conducted subgroup analyses stratified by sex, age, BMI, and DM status (Fig. 3). These results suggested that the TyG-BMI index and METS-IR were more suitable for predicting clinical worsening in IPAH patients with age ≥ 30. However, the results indicated a significant interaction between the age subgroups and the impact of METS-IR on the incidence of adverse outcomes (P for interaction = 0.007).

### Incremental predictive performance of IR indices in the risk assessment of clinical worsening

**Fig. 4:**
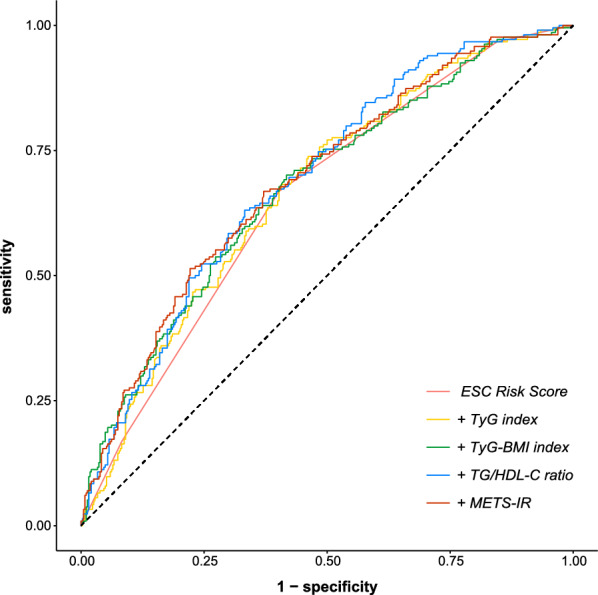
ROC curves of insulin resistance indices as a marker to predict clinical worsening based on ESC risk score. *ESC* European Society of Cardiology, *METS-IR* metabolic score for insulin resistance, *ROC* receiver operator characteristic, *TG/HDH-C* triglyceride to high-density lipoprotein cholesterol ratio, *TyG* Triglyceride and glucose, *TyG-BMI* triglyceride glucose-body mass index

ROC curves were constructed to explore the predictive ability of the ESC risk stratification and the ESC model plus each of the four IR indices for clinical worsening (Fig. 4). The C-statistics, NRI, and IDI are presented in Table 4. Addition of the TyG-BMI index, TG/HDL-C ratio, and METS-IR had significant incremental effects on the C-statistics of the ESC model (P < 0.05). However, only addition of the TG/HDL-C ratio and METS-IR significantly improved the reclassification and discrimination ability beyond that achieved by the ESC risk stratification.

**Table 4:** Improvement in discrimination and risk reclassification for clinical worsening after adding insulin resistance indices

Model	C-statistic (95% CI)	*P* value	NRI (95% CI)	*P* value	IDI (95% CI)	*P* value
ESC risk score	0.655 (0.614, 0.697)	Ref	Ref		Ref	
+ TyG index	0.670 (0.626, 0.714)	0.102	0.121 (−0.046, 0.287)	0.155	0.003 (−0.002, 0.008)	0.184
+ TyG-BMI index	0.677 (0.632, 0.721)	0.039	0.109 (−0.057, 0.276)	0.199	0.012 (0.003, 0.021)	0.009
+ TG/HDL-C ratio	0.690 (0.647, 0.733)	0.001	0.172 (0.009, 0.336)	0.039	0.017 (0.006, 0.029)	0.003
+ METS-IR	0.689 (0.645, 0.732)	0.005	0.216 (0.050, 0.381)	0.011	0.022 (0.010, 0.035)	< 0.001

*CI* confidence interval, *ESC* European Society of Cardiology, *IDI* integrated discrimination improvement, *METS-IR* metabolic score for insulin resistance, *NRI* net reclassification improvement, *TG/HDH-C* triglyceride to high-density lipoprotein cholesterol ratio, *TyG* Triglyceride and glucose, *TyG-BMI* triglyceride-glucose-body mass index

## Discussion

To the best of our knowledge, no previous study has attempted to compare the association between different IR indices and the severity of IPAH and its adverse outcomes. Furthermore, this study offers a fresh perspective and novel evidence for risk stratification in patients with IPAH. We found that various IR indices were correlated positively with NT-proBNP, TAPSE/sPAP, and RAP but correlated negatively with peakVO_2_, cardiac index, SVI, and S_V_O_2_. Moreover, elevated TG/HDL-C ratio and METS-IR levels were observed in high to intermediate-high risk IPAH patients as compared to low to intermediate-low risk patients. After accounting for confounding factors, TyG-BMI index and METS-IR were identified as independent predictors of mortality, lung transplantation, and heart failure-related readmission in patients with IPAH. TG/HDL-C ratio and METS-IR enhanced the predictive capability of the ESC-recommended risk stratification tool for adverse outcomes. In summary, IR indices, particularly METS-IR, can serve as crucial indicators for assessing the severity and clinical outcomes in patients with IPAH. Moreover, they hold significant value for refining guideline-recommended risk stratification tools.

Previous investigations of the correlation between IR and PAH have predominantly focused on the TG/HDL-C ratio. Brunner et al.[32] observed IR and insulin-sensitive patients with PAH and compared their TG/HDL-C ratios. They discovered that the IR group exhibited a worse New York Heart Association classification, mitral inflow E wave velocity, E/A ratio, and lateral mitral valve E' velocity. These findings suggest a connection between IR, poor functional classification, and left ventricular diastolic function in patients with PAH. Jonas et al.[33] determined that IPAH patients with TG/HDL-C > 3 experienced elevated levels of interleukin (IL)-1β, monocyte chemotactic protein-1, and IL-6 in comparison to IPAH patients with TG/HDL-C ≤ 3. These elevated levels indicate the presence of a systemic inflammatory state. Moreover, a retrospective cohort study conducted in Poland demonstrated that the TG/HDL-C ratio was higher in patients than in healthy individuals [34]. After adjusting for variables such as age, sex, and PAH severity, a higher TG/HDL-C ratio was significantly associated with overall mortality in patients with IPAH. However, the number of patients included in these studies was small, different IR indices were not evaluated or compared, and the impact of IR status on hemodynamic parameters in patients with PAH was not investigated. Therefore, the present study investigated the association between the four IR indicators and functional status, ultrasound indicators, hemodynamic parameters, and adverse outcomes in patients with IPAH.

A growing body of clinical evidence indicates that multiple metabolic factors are associated with a poor prognosis in PAH. Elevated blood glucose levels have been independently correlated with overall mortality in patients with IPAH [34]. Additionally, for patients with IPAH without DM, those with HbA1c < 5.7% have a substantial advantage in terms of 5-year survival compared to patients with higher initial values. HbA1c levels at the time of PAH diagnosis serve as independent prognostic factors for long-term outcomes [35]. HDL-C possesses antioxidant and anti-inflammatory properties, which mitigate endothelial dysfunction and safeguard against pulmonary vascular dysfunction [36]. Heresi et al. [37] reported that lower HDL-C levels in patients were associated with increased mortality rates and clinical deterioration. Even after adjusting for cardiovascular risk factors, C-reactive protein, IR indices, and PAH severity, the HDL-C level remained a predictive marker of survival. Similarly, reduced levels of LDL-C, a recognized risk factor for CVDs, significantly predict mortality in patients with PAH [4]. Changes in glucose and lipid metabolism are frequently observed in obese patients. While some studies have reported the existence of an “obesity paradox,’’ which suggests an association between obesity and reduced mortality in patients with precapillary pulmonary hypertension [38], Weatherald et al. [39] discovered that obese patients with PAH had a shorter 6MWD and higher PAWP than non-obese patients with PAH. They also noted an interaction between age and obesity, with a substantial increase in mortality among patients with morbid obesity and who were aged < 65 years. This study underscores the importance of weight management in young PAH patients with morbid obesity.

Our research demonstrated that the METS-IR was an independent predictor of the severity of the ailment and significant endpoint incidents in patients with IPAH, while also enhancing the predictive efficacy of the ESC risk stratification tool for long-term patient outcomes. This could be attributed to the comprehensive consideration of three factors by the METS-IR: blood glucose level, lipid metabolism, and obesity. In addition, the METS-IR performed better than other IR indicators for other cardiovascular conditions. For example, when compared with the TyG index and the TG/HDL-C ratio, only METS-IR exhibited a significant correlation with prehypertension among the Chinese population with normal plasma glucose levels [40], thereby better distinguishing prehypertension [41]. Furthermore, only the METS-IR demonstrated autonomous capability in predicting the occurrence of asymptomatic adult coronary artery calcification in populations without CVDs [42]. Additionally, the METS-IR outperformed the TyG index and homeostatic model assessment for IR in predicting major adverse cardiovascular events in patients with DM, indicating an outstanding ability to identify major adverse cardiac events, as evidenced by the highest area under the curve value [43]. Taken together, the METS-IR may be particularly useful for evaluating the severity and prognosis in patients with CVDs.

As an emerging biomarker, the METS-IR offers several clinical benefits. Unlike the hyperinsulinemic–euglycemic clamp technique, the METS-IR only requires data collection on fasting blood glucose, blood lipids, height, and weight for computation. Thus, the METS-IR is a convenient, cost-effective, and feasible method for assessing the IR status in patients with IPAH. Previous studies have demonstrated the significance of evaluating the prevalence of DM in determining the severity and prognosis of IPAH. Whitaker et al. [44] found that, in patients with combined PAH and DM, after adjusting for age, sex, PVR, and targeted drug use, the pulmonary arteries were stiffer, pulmonary artery capacity was lower, and right ventricular wall thickness measured via ultrasound was greater than that in PAH patients without DM. A retrospective study conducted on a cohort of 9017 IPAH patients from the National Health Insurance Service in Korea revealed that IPAH patients with DM exhibited an HR of 1.29 (95% CI 1.17–1.42), thus indicating DM as a risk factor for IPAH [45]. Jonas et al. [6] conducted a matched analysis involving 136 patients each with IPAH with and without DM and revealed that the former cohort exhibited higher RAP, mPAP, PVR, and overall mortality than did the latter. However, it should be acknowledged that the Chinese population presents a younger age of onset and a lower proportion of DM than do populations in other countries [46, 47]. Therefore, assessing the presence of DM alone is insufficient to evaluate patients with IPAH. By introducing various indices of IR, this study demonstrated that METS-IR can independently predict both the severity of IPAH and major adverse events. Notably, the predictive value of METS-IR for adverse events remained unaffected by the presence of DM in patients with IPAH, highlighting the equal importance of assessing IR levels in diabetic and non-diabetic patients with IPAH.

However, the precise physiological mechanisms underlying the relationship between IR and PAH remain unclear. Ongoing research primarily encompasses various facets, such as the activation of inflammation, mutation in the bone morphogenic protein receptor type 2 (BMPR2), deficiency in the peroxisome proliferator-activated receptor gamma (PPARγ), and reduced levels of adiponectin. IR can instigate an upsurge of inflammatory cytokines associated with obesity, including IL-1β, IL-2, IL-4, IL-5, IL-6, IL-8, tumor necrosis factor-α, and interferon, which have been robustly linked to the development of PAH [48, 49]. In the IR state, monocyte-derived inflammatory macrophages expressing C–C chemokine receptor 2 (CCR2 +) are notably increased, coupled with increased expression of nucleotide-binding domain, leucine-rich-containing family, pyrin domain-containing-3 (NLPR3) inflammasome, leading to mitochondrial dysfunction. This phenomenon has been observed in a monocrotaline-induced rat model, in which the inhibition of NLPR3 resulted in an improvement in right heart function in PAH rats [50]. Hemnes et al. [51] conducted proteomic and metabolomic analyses of PAH patients with IR and matched control groups, which identified significant increases in proteins related to insulin metabolism and oxidized LDL receptor 1 (OLR1) in PAH patients. Immunostaining of lung tissue lesions in patients with PAH revealed enhanced OLR1 expression, implying the potential involvement of OLR1 in the pro-inflammatory phenotype of PAH. Mutations in BMPR2 contribute to reduced BMPR2 expression, which is a key pathophysiological mechanism in the development of PAH [52]. West et al. [53] performed a metabolomic analysis of mice with BMPR2 mutations, uncovering substantial weight gain and IR as compared to the control group, suggesting that IR manifested as an early characteristic of BMPR2 mutations in mice. PPARγ is a nuclear receptor and transcription factor governing fatty acid and glucose metabolism, and its deficiency is considered to be a trigger for IR [54]. Adiponectin, a hormone that regulates fat and glucose metabolism, exhibits anti-inflammatory properties, improves insulin sensitivity, and reverse IR [55]. In a high-fat diet-induced PAH mouse model, treatment with pioglitazone reversed the diminished expression of adiponectin and PPARγ in pulmonary arteries, thereby ameliorating IR and pulmonary artery pressure [56]. Studies have identified decreased levels of PPARγ and adiponectin in PAH patients, potentially offering insight into the mechanisms linking IR to PAH development [57]. In addition, patients with PAH often have varying degrees of cardiac dysfunction and microcirculation dysfunction, which is an important factor aggravating IR [58]. According to Swan et al., chronic heart failure independently predicts impaired insulin sensitivity when comparing coronary heart disease patients with and without concurrent chronic heart failure [59]. The reduced physiological functions in heart failure patients, such as glucose and insulin transport to skeletal muscle, diffusion through the endothelium, signal transduction processes, and glucose uptake, may contribute to this phenomenon [60]. Research has demonstrated that microcirculation disorders are associated with IR development. As insulin sensitivity decreases, the utilization of nitric oxide also diminishes, resulting in increased levels of intracellular Ca^2+^ and enhanced Ca^2+^ sensitivity in vascular smooth muscle cells. This cascade effect triggers local vasoconstriction, contributing to microcirculation disorders and subsequent inadequate tissue perfusion [61]. Consequently, the lowered energy state leads to alterations in calcium metabolism and cell apoptosis, exacerbating IR [62].

## Limitations

This study had the following limitations. First, this was a retrospective study. Nevertheless, to the best of our knowledge, no previous study with a large sample size has compared the applicability of IR indices in patients with IPAH. Second, due to the lack of dynamic data on blood glucose, lipid levels, and risk stratification, it is not possible to dynamically evaluate the effect of IR indices on the severity and prognosis of IPAH. Third, despite adjusting for several confounding factors, such as age, sex, DM, and WHO-FC, the existence of other potential confounding factors, such as dietary habits, physical exercise, and sleep cannot be ruled out. In future investigations, it will be imperative to conduct further assessments and adjust for these additional confounding factors.

## Conclusions

This study found that higher IR indices were associated with a higher likelihood of a non-low-risk status in patients with IPAH. Various IR indices were correlated with cardiac function, echocardiographic indicators, and hemodynamic parameters. Importantly, we discovered that TyG-BMI index and METS-IR independently predicted clinical worsening in patients. The addition of the METS-IR to the ESC risk score enhanced the latter’s predictive capability. Consequently, the METS-IR can be a dependable and convenient predictor of the severity and long-term adverse outcomes in patients with IPAH and can be used to optimize the risk stratification tools recommended in the guidelines.

## Supplementary Information

**Additional file 1: Table S1. **Definition of insulin resistance indexes. **Table S2.** Variables and cut-off values used for four-strata European Society of Cardiology risk score. **Figure S1.** The flowchart of study participants. BMI, body mass index; CW, clinical worsening; FPG, fasting plasma glucose; HDL-C, high-density lipoprotein cholesterol; IPAH, Idiopathic pulmonary arterial hypertension; TG, triglycerides.

## Data Availability

The datasets used and analysed during the current study are available from the corresponding author on reasonable request.

## Abbreviations

ALT: Alanine aminotransferase
AST: Aspartate aminotransferase
BMI: Body mass index
CI: Confidence interval
CPET: Cardiopulmonary exercise testing
CVDs: Cardiovascular diseases
CW: Clinical worsening
DL_CO_: Carbon monoxide diffusing capacity
DM: Diabetes mellitus
ESC: European Society of Cardiology
FEV1: Forced expiratory volume in one second
FPG: Fasting plasma glucose
HbA1c: Hemoglobin A1c
HDL-C: High-density lipoprotein cholesterol
HR: Hazard ratio
IDI: Integrated discrimination improvement
IPAH: Idiopathic pulmonary arterial hypertension
IR: Insulin resistance
LA: Left atrium dimension
LDL-C: Low-density lipoprotein cholesterol
ln: Logarithmically transformed
LVED: Left ventricular end-diastolic diameter
METS-IR: Metabolic score for insulin resistance
mPAP: Mean pulmonary arterial pressure
6MWD: 6-Min walk distance
NRI: Net reclassification improvement
NT-proBNP: N-terminal pro-brain natriuretic peptide
PAH: Pulmonary arterial hypertension
PAWP: Pulmonary arterial wedge pressure
peak VO_2_: Peak oxygen uptake
PFT: Pulmonary function test
PVR: Pulmonary vascular resistance
RAP: Right atrial pressure
RVED: Right ventricular end-diastolic diameter
sPAP: Systolic pulmonary arterial pressure
SVI: Stroke volume index
S_V_O_2_: Mixed venous oxygen saturation
TAPSE: Tricuspid annular plane systolic excursion
TG/HDH-C: Triglyceride to high-density lipoprotein cholesterol ratio
TyG: Triglyceride and glucose
TyG-BMI: Triglyceride-glucose-body mass index
WHO-FC: World Health Organization functional class
