# Correction: Association of non-insulin-based insulin resistance indices with disease severity and adverse outcome in idiopathic pulmonary arterial hypertension: a multi-center cohort study

**DOI:** 10.1186/s12933-025-02792-8

**Published:** 2025-06-09

**Authors:** Sicheng Zhang, Luyang Gao, Sicong Li, Manqing Luo, Lichuan Chen, Qunying Xi, Zhihui Zhao, Qing Zhao, Tao Yang, Qixian Zeng, Xin Li, Zhihua Huang, Anqi Duan, Yijia Wang, Qin Luo, Yansong Guo, Zhihong Liu

**Affiliations:** 1 Center for Respiratory and Pulmonary Vascular Diseases, National Center for Cardiovascular Diseases, Fuwai Hospital, Chinese Academy of Medical Sciences and Peking Union Medical College, No. 167, Beilishi Road, Beijing, 100037 Xicheng China; 2 Department of Cardiology, Fujian Provincial Hospital, Shengli Clinical Medical College of Fujian Medical University, No. 134, East Street, Gulou District, Fuzhou, 350001 Fujian China; 3 Center for Pulmonary Vascular Diseases, Fuwai Hospital, Chinese Academy of Medical Sciences, Shenzhen, No. 12, Langshan Road, Shenzhen, 518057 Nanshan China

**Correction to: Cardiovascular Diabetology (2024) 23:154** 10.1186/s12933-024-02236-9

In the original publication of this article [1], the'Ethics' statement was incorrectly given as'The study protocol conformed to the ethical guidelines of the Declaration of Helsinki and was revised and approved by the local ethics committee' but should have been' The study protocol conformed to the ethical guidelines of the Declaration of Helsinki and was revised and approved by the Ethics Committee of Fuwai Hospital, Chinese Academy of Medical Sciences (Approval Number: 2022-1739)'.
