# Supplementary material for: Association of non-insulin-based insulin resistance indices with disease severity and adverse outcome in idiopathic pulmonary arterial hypertension: a multi-center cohort study
Source: Cardiovasc Diabetol. 2024 May 3;23:154. doi: 10.1186/s12933-024-02236-9 (PMC11069206; doi:10.1186/s12933-024-02236-9)
Supplement: Supplementary file 1 — Additional file 1: Table S1. Definition of insulin resistance indexes. Table S2. Variables and cut-off values used for four-strata European Society of Cardiology risk score. Figure S1. The flowchart of study participants. BMI, body mass index; CW, clinical worsening; FPG, fasting plasma glucose; HDL-C, high-density lipoprotein cholesterol; IPAH, Idiopathic pulmonary arterial hypertension; TG, triglycerides. [file 12933_2024_2236_MOESM1_ESM.docx]

**Additional file 1**

**Table S1. Definition of insulin resistance indexes.**

| **Variables** | **Formulas** |
| --- | --- |
| TyG index | TyG index = Ln (TG × FPG / 2) |
| TyG-BMI index | TyG-BMI index = Ln (TG × FPG / 2) × BMI |
| TG/HDL-C ratio | TG/HDL-C ratio = TG / HDL-C |
| METS-IR | METS-IR = Ln (2 × FPG + TG) × BMI / Ln (HDL-C) |

BMI (kg/m2), FBG (mg/dl), TG (mg/dl), and HDL (mg/dl) are used to in the formulas above.

Abbreviations: BMI, body mass index; FPG, fasting plasma glucose; HDL-C, high-density lipoprotein cholesterol; METS-IR, metabolic score for insulin resistance; TG, triglycerides; TG/HDH-C, triglyceride to high-density lipoprotein cholesterol ratio; TyG, Triglyceride and glucose; TyG-BMI, triglyceride glucose-body mass index.

**Table S2. Variables and cut-off values used for four-strata European Society of Cardiology risk score.**

| **Determinants of prognosis** | **Low risk** | **Intermediate-low risk** | **Intermediate-high risk** | **High risk** |
| --- | --- | --- | --- | --- |
| Points assigned | 1 | 2 | 3 | 4 |
| WHO-FC | I or II | - | III | IV |
| 6MWD, m | > 440 | 320-440 | 165-319 | < 165 |
| BNP, ng/L or  NT-proBNP, ng/L | < 50  < 300 | 50-199  300–649 | 200-800  650-1100 | > 800  > 1100 |

Risk is calculated by dividing the sum of all grades by the number of variables and rounding to the next integer.

Abbreviations: 6MWD, 6-minute walking distance; BNP, brain natriuretic peptide; NT-proBNP, N-terminal pro-brain natriuretic peptide; WHO-FC, World Health Organization functional class.

**
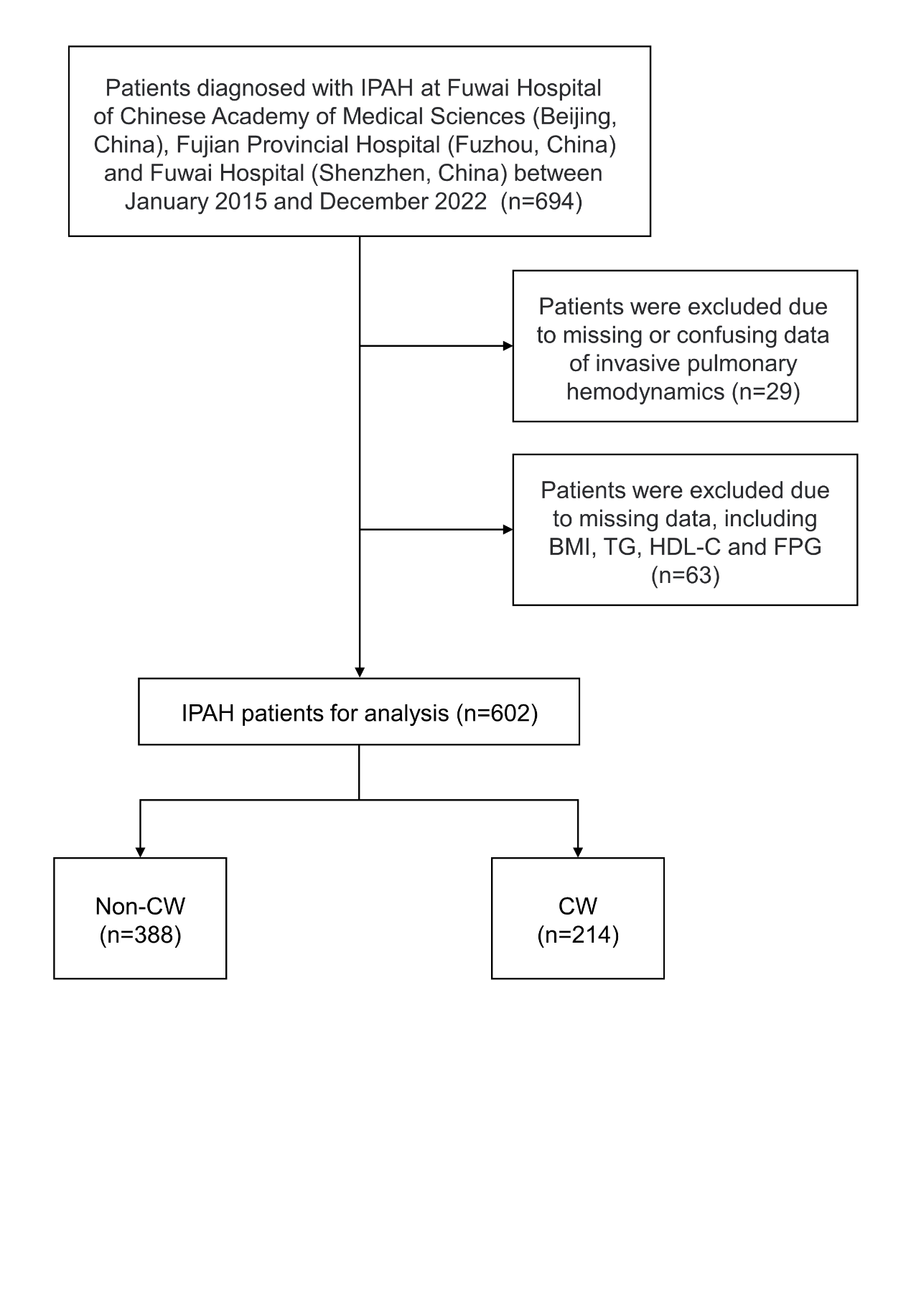
**

**Figure S1. The flowchart of study participants.**

Abbreviations: BMI, body mass index; CW, clinical worsening; FPG, fasting plasma glucose; HDL-C, high-density lipoprotein cholesterol; IPAH, Idiopathic pulmonary arterial hypertension; TG, triglycerides.
